# An open-source replication for fast and accessible light propagation modeling in brain tissue

**DOI:** 10.1371/journal.pone.0330681

**Published:** 2025-09-02

**Authors:** David Berling, Jan Střeleček, Tomáš Iser, Ján Antolík

**Affiliations:** Faculty of Mathematics and Physics, Charles University, Prague, Czechia; Radiation Application Research School, NSTRI, IRAN, ISLAMIC REPUBLIC OF

## Abstract

Light simulations hold great potential for advancing optical techniques in neuroscience. They facilitate the in-silico refinement of optical stimulator designs and enable simulations of optical recordings from computational brain models, aiding neuroscience in forming a mechanistic understanding of brain circuitry. However, many published light models are inaccessible due to unavailable source code and documentation or are impractical due to excessive computational demands. To address these challenges, we replicate and enhance the efficient and accurate light simulation model by Yona et al. [1], which was previously available only in compiled form accompanied by sparse documentation. In this work, we resolve ambiguities in the original model, correct errors that caused discrepancies between simulations and published results, improve computational efficiency by an order of magnitude, and open-source all the resulting code and detailed documentation. These enhancements enable simulations of cortical volumes exceeding $1\;{\text{mm}}^{3}$ to run in seconds on standard laptop hardware. Our model software provides an accessible, adaptable, and rapid light simulation tool, which adheres to FAIR principles to ensure future-proof and broad utility for the neuroscience community.

## Introduction

Optical techniques, such as optogenetic stimulation and calcium imaging, are key tools in modern neuroscience for manipulating and recording neuronal signals in the brain [2, 3]. Accurate simulations of light spread in cortical tissue can support the application of these techniques through identifying the spatial reach of optogenetic stimulation inside cortical tissue or the spatial blurring of optically recorded neuronal activation. These simulations can accelerate the development of new experimental setups by allowing researchers to screen a variety of designs in-silico, reducing the need for time-consuming and expensive real-world testing.

Yona et al. [1] suggested a computationally efficient light-propagation model based on a beam-spread-function approach as an alternative to commonly-used Monte Carlo light simulations, which are computationally exceedingly expensive. They validated their model against experimental data and benchmarked it against a Monte Carlo model, demonstrating high prediction accuracy and superior computational performance. However, the published software has major issues: First, constrained parameter settings restrict the simulated volume to 700 μm vertically and 400 μm horizontally, preventing light simulation in deeper cortical layers and larger horizontal distances. Second, simulation results obtained through the software are inconsistent with published data at greater radial distances and depths. Third, the lack of source code makes adapting the model to other stimulator designs impossible. Finally, the original article and model documentation lack a description of critical implementation details, making the model’s predictions non-transparent and hindering replication to verify its validity.

Given the model’s innovative and flexible approach, which enables efficient and accurate modeling of light transport through cortical tissue, we re-implemented their method from scratch, enhancing computational efficiency to support simulation of larger cortical volumes ($\geq 1\;{\text{mm}}^{3}$). In our replication, we identified and resolved ambiguity in key aspects of model implementation, and reproduced a corrected error in the original MATLAB software, which causes the observed inconsistency between results published and simulated with the original software.

Our replication is published under the GPL-3.0 license, listed in the Python Package Index (https://pypi.org/project/bsf-light/), and adheres to the FAIR principles for research software.

## Materials and methods

### Description of direct and scattered light with an infinitesimal pencil beam

Yona et al.’s approach [1] utilizes an infinitesimal light beam, called pencil beam, which represents photons sent from an infinitesimal point along one direction (here *z*-direction). Loss of photons from this beam due to scattering or absorption is a probabilistic process which enters the expression for the contribution of direct photons to light intensity in a point in cylindrical coordinates (*ρ*,*z*) as

$$I_{\text{pencil}}^{\text{direct}}(\rho ,z)=\exp(-(\mu_{a}+\mu_{s})\cdot z)\;\delta (\rho ),$$ (1)

where $\mu_{s}$ and $\mu_{a}$ describe the coefficients for scattering and absorption, which quantify the probability of scattering and absorption per unit length, and *δ* the Dirac delta function.

The contribution of scattered photons to the light intensity can be described with a beam-spread-function approach, which describes how many scattered photons traverse a point (*ρ*, *z*), given their multipath time (*τ*), the time they have for scattering to reach this point:

$$i_{\text{pencil}}^{\text{scatter}}(\tau ,\rho ,z)=(1-\exp(\mu_{s}\cdot z))\cdot \exp(-\mu_{a}\cdot (z+c\cdot \tau ))\cdot G(\tau ,z)\cdot h(\tau ,\rho ,z),$$ (2)

where the factors describe (in order of appearance) the fraction of scattered out of all photons, the probability to remain in the beam despite absorption, the distribution function of multi-path time *G*, and a spatial-angular distribution function *h*. Further, *c* describes the speed of light in the medium. *G* and *h* are defined as follows:

$$G(\tau ,z)=\frac{\mu_{\tau }}{\sigma_{\tau }^{2}\Gamma (\frac{\mu_{\tau }^{2}}{\sigma_{\tau }^{2}})}\cdot {\left(\frac{\mu_{\tau }\tau }{\sigma_{\tau }^{2}}\right)}^{\mu_{\tau }^{2}/\sigma_{\tau }^{2}-1}\cdot \exp\left(-\frac{\mu_{\tau }\tau }{\sigma_{\tau }^{2}}\right),$$ (3)

$$h(\tau ,\rho ,z)=\frac{3}{4\pi \tau cz}\cdot \exp\left(-\frac{3}{4}\frac{\rho^{2}}{\tau cz}\right).$$ (4)

*G* involves the first and second moment of the multi-path time, $\mu_{\tau }$ and $\sigma_{\tau }$, respectively, which both depend on *z*. Yona et al. [1] refer for their derivation to McLean et al. [4], who offer different ways for their calculation, which we cover separately in the following section.

To arrive at an expression for the contribution of scattered photons to intensity in a point (*ρ*,*z*), Eq 2 needs to be integrated over multi-path time:

$$I_{\text{pencil}}^{\text{scatter}}(\rho ,z)=\int i_{\text{pencil}}^{\text{scatter}}(\tau ,\rho ,z)d\tau .$$ (5)

### Calculation of the moments of multi-path time *τ*

Yona et al. [1] refers to McLean et al. [4], which quotes precise derivations of the first ($\mu_{\tau }$) and second moment ($\sigma_{\tau }$) by Lutomirski et al. [5] (cf. Eqs 4 and 5 in McLean et al.):

$$\frac{\mu_{\tau }}{z/c}=1-\frac{1-\exp(-\mu_{s}zv)}{\mu_{s}zv},$$ (6)

where

v=1−⟨cos(θ)⟩, (7)

which is related to the anisotropy index *g*, describing scattering directionality, being defined as the first moment of the cosine of the scattering angle (g=⟨cos(θ)⟩). For the second moment they provide:

$$\frac{\sigma_{\tau }^{2}}{z^{2}/c^{2}}=\frac{2}{3}\frac{\left(w^{2}-3wv)[\exp(-\mu_{s}zv)-1+\mu_{s}zv]+2v^{2}[\exp(-\mu_{s}zw)-1+\mu_{s}zw\right]}{\mu_{s}^{2}z^{2}wv^{2}(w-v)}\;\;-{\left[\frac{1-\exp(-\mu_{s}zv)}{\mu_{s}zv}\right]}^{2},$$ (8)

where

$$w=\frac{3}{2}\cdot (1-\langle \cos(\theta {)}^{2}\rangle ),$$ (9)

with the second moment of the cosine of the scattering angle, $\langle \cos(\theta {)}^{2}\rangle$.

McLean et al. further give approximations for the calculation of the first and second moments by Lutomirski et al. (Eqs 4, 5 and Table 1 in McLean et al.):

$$\frac{\mu_{\tau }}{z/c}=\frac{1}{4}\mu_{s}z\langle \theta^{2}\rangle ,$$ (10)

$$\frac{\sigma_{\tau }^{2}}{z^{2}/c^{2}}=\frac{1}{12}\mu_{s}z\langle \theta^{4}\rangle +\frac{1}{24}(\mu_{s}z{)}^{2}\langle \theta^{2}\rangle^{2},$$ (11)

where $\langle \theta^{2}\rangle$ is the first, and $\langle \theta^{4}\rangle$ is the second moment of the squared scattering angle. And further provide approximations by van de Hulst and Kattawar [6] (Table 1 in McLean et al. [4]):

$$\frac{\mu_{\tau }}{z/c}=\frac{1}{12}\mu_{s}z\langle \theta^{2}\rangle ,$$ (12)

$$\frac{\sigma_{\tau }^{2}}{z^{2}/c^{2}}=\frac{7}{720}(\mu_{s}z{)}^{2}\langle \theta^{2}\rangle^{2}.$$ (13)

**Table 1:** Chosen default parametrization of the model.

Parameter	Value	Unit	Description
c_0	0.299792458	μm/fs	Speed of light in vacuum
**Tissue properties**			
ntissue	1.36		Refractive index of cortical tissue
mu_a	0.00006	$\;\mu {\text{m}}^{-1}$	Absorption coefficient
mu_s	0.0211	$\;\mu {\text{m}}^{-1}$	Scattering coefficient
g	0.86		Anisotropy factor
**Optical fiber**			
NA	0.37		Numerical aperture of the fiber
opt_radius	100	μm	Radius of the optical fiber
**Final volume**			
xymax	700	μm	Maximum xy dimensions
dxy	5	μm	Step size in the xy plane
zmax	700	μm	Maximum z dimension
dz	5	μm	Step size in the z direction
**Calculation of scattered pencil beam**			
rho_exp_min	1	μm	Minimum value of radial log.-sampling (will be subtracted to start from 0)
n_rho_smpls	20		Number of radial samples
tau_min	5	fs	Minimum time for multipath-time integral
tau_max	10000	fs	Maximum time for multipath-time integral
n_tau_smpls	100		Number of time samples for multipath-time integral
mu_tau	"eq4"		Equations to calculate the moment, "eq4" refers to precise calculation of *μ* and relating *σ* to *μ* (based on approximations by Lutomirski et al., Eqs 10 and 11)
**Angular convolution of scattered pencil beam**			
nsteps_theta	24		Number of angular steps in *θ*
nsteps_phi	24		Number of angular steps in *ϕ*
**Disk convolution**			
dxy_direct_disk	3	μm	Stepsize for disk convolution of direct light
dxy_scattered_disk	10	μm	Stepsize for disk convolution of scattered light

In the approximations, $\langle \theta^{2}\rangle$ can be related to the anisotropy index *g* via $\langle \theta^{2}\rangle =2(1\;-\;g)$ (comment below Table 1 in McLean et al.).

### Construction of a light cone from pencil beams

A light cone emitted from an infinitesimal point can be represented as the superposition of an infinite number of pencil beams, uniformly distributed over a spherical cap defined by the fiber’s opening angle. This composition can be expressed as a convolution over the azimuthal (*ϕ*) and polar (*θ*) angles, which describe the opening of the light cone in spherical coordinates (*r*, *ϕ*, *θ*):

$$I_{\text{cone}}[\rho (r,\phi ,\theta ),z(r,\phi ,\theta )]=\int d\phi^{\prime }\int d\theta^{\prime }\;r^{2}\sin\theta^{\prime }I_{\text{pencil}}[\rho (r,\phi -\phi^{\prime },\theta -\theta^{\prime }),z(r,\phi -\phi^{\prime },\theta -\theta^{\prime })].$$ (14)

The integration over $\phi^{\prime }$ covers the full circle ([0,2π]) and over $\theta^{\prime }$ spans from zero to the opening angle ($\left[0,\theta_{\text{div}}\right]$). Latter is given by $\theta_{\text{div}}=\sin^{-1}\left(\frac{NA}{n}\right)$, with the numerical aperture of the fiber, *NA*, which describes the light-gathering ability of an optical system, and the refractive index, *n*, capturing the ratio of the wavelength of light in vacuum to the wavelength in a medium, here, brain tissue.

For the direct light component, we derived following expression analytically:

$$I_{\text{cone}}^{\text{direct}}(\rho ,z)=\frac{\exp(-(\mu_{a}+\mu_{s})\cdot \sqrt{\rho^{2}+z^{2}})}{2\pi (1-\cos(\theta_{\text{div}}))\cdot (\rho^{2}+z^{2})}.$$ (15)

For the scattered component, we approximated the integral numerically using a Riemann sum:

$$I_{\text{cone}}^{\text{scatter}}[\rho (r,\phi ,\theta ),z(r,\phi ,\theta )]=\;\frac{\sum_{\phi^{\prime }}\sum_{\theta^{\prime }}\;I_{\text{pencil}}^{\text{scatter}}[\rho (r,\phi -\phi^{\prime },\theta -\theta^{\prime }),z(r,\phi -\phi^{\prime },\theta -\theta^{\prime })]r^{2}\sin\theta^{\prime }d\phi^{\prime }d\theta^{\prime }}{2\pi r^{2}}.$$ (16)

### Construction of the optical fiber beam from light cones

Finally, we arrive at an expression of the light emitted from an optical fiber by representing it as the composition of light cones emitted from infinitesimal points distributed over the fiber’s disk-shaped emission surface. We approximate this integral using a Riemann sum as

$$I_{\text{fiber}}[\rho (x,y),z]=\sum_{x^{\prime },y^{\prime }\in \vec{A}}\;\left(I_{\text{cone}}^{\text{direct}}+I_{\text{cone}}^{\text{scatter}}\right)[\rho (x-x^{\prime },y-y^{\prime }),z]\;dx^{\prime }dy^{\prime },$$ (17)

where the sum expands over all *x*^′^ and $y^{\prime }$ contained in the fiber’s emission surface $\vec{A}$. Note that the sum may be computed separately at individually chosen step sizes ($dx^{\prime }$, $dy^{\prime }$) for the direct and scattered light component.

### Parametrization of numerical integrals and sampling

Direct and scattered photon contributions were calculated separately and combined after the disk convolution. Direct contributions of the pencil beam were analytically convolved over the fiber’s opening angle, sampled uniformly along radial ([0, 700; Δ=5] μm) and depth ([1, 700; Δ=5] μm) directions, and numerically convolved over the disk-shaped emission surface using linear interpolation between sampling points. The convolution was performed by summing points within a 100 μm radius at a stepsize of 3 μm in *x*/*y*-direction (Cartesian grid) as described by Eq 17. The convolution was calculated using only points with positive *y*-coordinate and multiplied by 2 to exploit symmetry and save computation time.

Scattered photon contributions of the pencil beam were logarithmically sampled along radial direction ([1 μm, 701 μm; n=20], using natural log.). For the sampling to start at zero, the start value was subtracted from the entire interval (final interval: [0, 700] μm). Contributions were sampled uniformly along depth direction ([1, 700, Δ=5] μm). Multipath time integration was carried out using logarithmic samples in time ([5 fs, 10000 fs; n=100], using natural log.). The scattered contributions were then convolved over the fiber’s opening (spherical) angle (Eq 16) using numerical integration with uniform angular samples along polar angle *θ* ([0, $\theta_{\text{div}}$]) and azimuthal angle *ϕ* ([0, 2π]), each using 24 steps. The angular convolution was performed with uniform sampling along radial ([0, 700; Δ=5] μm) and depth ([1, 700; Δ=5] μm) directions, using linear interpolation between sampling points of the pencil beam data. Finally, scattered contributions were convolved over the fiber’s disk-shaped emission surface as described for direct contributions before but with a step size of 10 μm.

## Results

The original article [1] outlines the ansatz for deriving the beam-spread function (Eqs 1–4 in this article) and provides a heuristic overview of the steps to calculate the light emission from an optical fiber:

1. Integration of the scattered pencil beam component over multi-path time,
2. performing an angular convolution of the combined (direct+scattered) pencil beam over the optical fiber’s opening angle to approximate a light cone.
3. Finally, spatially convolving the light cone over the disk-shaped emission surface of the optical fiber.

**Fig 1:**
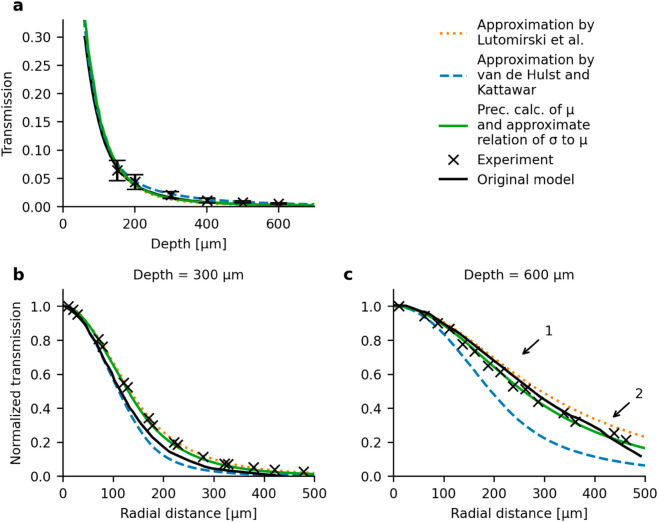
Parametrization of time dispersion moments for optimal transmission results. **(a)** Intensity of light transmission along depth for calculating moments of time dispersion according to approximations by Lutomirski et al. [5] (dotted orange), approximations by van de Hulst and Kattawar [6] (dashed blue), and using the precise calculation of $\mu_{\tau }$ (Eq 6) combined with relating $\sigma_{\tau }$ to $\mu_{\tau }$ based on the approximations by Lutomirski et al. (solid green). Experimental data and model predictions as originally published by Yona et al. [1] in black. **(b)** Intensity of light transmission normalized to intensity at the center of the beam at a depth of z=300 μm and **(c)** at a depth of z=600 μm. Color coding same as in (a). Arrow 1 indicates the point at which the original model (solid black) starts to deviate from the replication using the approximation by Lutomirski et al. (dotted orange). Arrow 2 highlights where the original model suddenly starts to decay more strongly than the replications and than what the experimental data would suggest. Parametrization of simulations shown in the figure: The precise calculation of $\mu_{\tau }$ and approximate relation of $\sigma_{\tau }$ to $\mu_{\tau }$ (solid green) refers to the default parametrization summarized in Table 1. The approximations by Lutomirski et al. (dotted orange) and van de Hulst and Kattawar (dashed blue) refer to the same parametrization but with the parameter "mu_tau" set to "table1_Lutomirski" and "table1_vandeHulst", respectively.

The first of these steps introduces ambiguity, as it requires parametrization of the time dispersion distribution *G*, which depends on the distribution’s first and second moments, $\mu_{\tau }$ and $\sigma_{\tau }$, which are not clearly defined. Yona et al. [1] reference McLean et al. [4] for their calculation, but McLean et al. provide three different sets of equations for their calculation. The first set is a precise derivation by Lutomirski et al. [5] (Eqs 6 and 8), which allows for calculation of the first moment $\mu_{\tau }$. However, it does not enable calculation of the second moment $\sigma_{\tau }$, as this depends on the second moment of the cosine of the scattering angle ($\langle \cos\theta^{2}\rangle$), which we could neither generally derive, nor find in the articles by Yona et al. or McLean et al.. An analogous issue applies to the second set of equations, an associated approximation of the moments also by Lutomirski et al. (Eqs 10 and 11), which depend on the second moment of the squared scattering angle ($\langle \theta^{4}\rangle$) that we could not resolve. In contrast, the third set, an approximation of the moments by van de Hulst and Kattawar (Eqs 12 and 13), does not depend on the second moment of the squared scattering angle, and enables relating $\sigma_{\tau }$ to $\mu_{\tau }$ through a constant ratio: $\sigma_{\tau }=(1/12)\;\cdot \;\mu_{\tau }/\sqrt{7/720}$ (see Eqs 12, 13). We used this approach to calculate light transmission, comparing it to experimental data by Yona et al., see Fig 1a–1c, dashed blue curve and black crosses, respectively, finding matching results for transmission over depth (Fig 1a), but underestimation of light spreading for transmission over radial distance (Fig 1b, 1c).

Inspired by the idea of relating $\sigma_{\tau }$ and $\mu_{\tau }$ to avoid the unknown second moment of the squared scattering angle, we tested whether an approximate relation derived based on Lutomirski et al.’s approximations could succeed (Eqs 10 and 11). Assuming the second moment of the squared scattering angle *θ* in Eq 11 to be zero under the small-angle approximation, gives a ratio of $\sigma_{\tau }=(1/4)\;\cdot \;\mu_{\tau }/\sqrt{1/24}$. This approach also achieved matching transmission over depth, see Fig 1a, dotted orange, but additionally, achieved a closer match for transmission over radial distance compared to the previous, see Fig 1b, 1c, dotted orange; still overestimating transmission at large depths and radial distances, see Fig 1c. Finally, we combined the precise calculation of the first moment (Eq 6) with the second moment calculated with the constant ratio between $\mu_{\tau }$ and $\sigma_{\tau }$ based on Lutomirski et al.’s approximations (Eqs 10 and 11). This approach also produced matching transmission results over depth, see Fig 1a, solid green; and was in strongest agreement with the experimental data on radial transmission among all, see Fig 1b, 1c, solid green.

Despite the strong agreement of this approach with experimental data, neither this nor both other parameterizations of the time dispersion moments reproduced the original model predictions exactly: They agreed for transmission over depth (Fig 1a), but disagreed for radial transmission at z=300 μm (Fig 1b), and only the replication using Lutomirski et al.’s approximations matched partially at a depth of z=600 μm for radii under ρ=200 μm, see Fig 1c (dotted orange vs. black curves). Beyond ρ=200 μm the original model decays slightly more than the replication using Lutomirski et al.’s approximations (see arrow ‘1’ in Fig 1c). At around ρ=400 μm the original model shows a sudden and even stronger decay, see arrow ‘2’ in Fig 1c. The original model predictions hence not only deviate more than the optimal replication version from the original experimental data but they also cannot clearly be attributed to any of the alternative replications.

In summary, we could not finally resolve which parametrization of the time dispersion moments was used by Yona et al.. Our replication results agreed with the original study’s model predictions most closely when the time dispersion moments were calculated using the approximations by Lutomirski et al. (Eqs 10 and 11). However, we found that the parametrization of the time dispersion moments using the precise derivation of the first moment $\mu_{\tau }$ according to Eq 6, combined with an approximate relation of the second moment $\sigma_{\tau }$ to the first $\mu_{\tau }$, achieves the most optimal agreement of model predictions with experimental data. We therefore selected this parametrization as the default for our model, which is summarized in Table 1.

### Reproduction of a prediction error in the original model software

**Fig 2:**
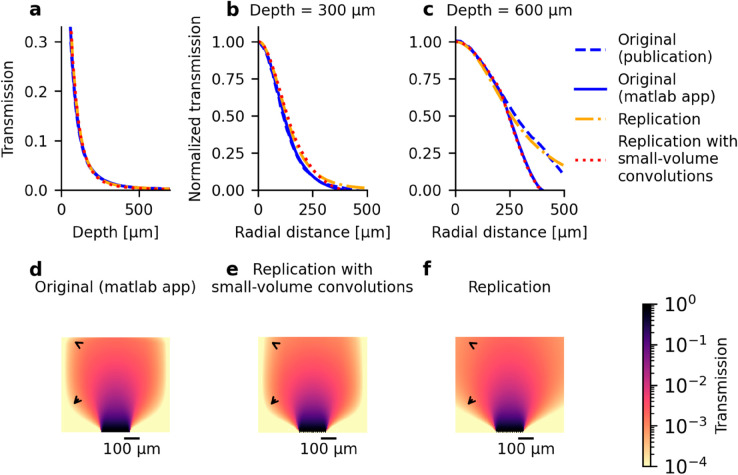
Adequate sampling volume for spatial convolutions avoids prediction error occurring in the original software. **(a)** Light transmission along depth at beam center as published originally (dashed blue), as simulated with the original MATLAB software (solid blue), as simulated with our replicated model using adequate sampling volume during spatial convolutions (dash-dotted orange) and using a reduced sampling volume (dotted red). All methods provide identical output. **(b)** Light transmission along radial direction normalized to beam center at a depth of z=300 μm and **(c)** at a depth of z=600 μm. Color coding same as in (a). **(d)** 2D light transmission profile along radial and depth direction as simulated with the original MATLAB software. The data underestimate light intensity at around 350 μm distance from beam center as visually apparent by an edge. Further, underestimation of light intensity is even more pronounced at the profile’s ‘corners’ as marked by arrows. **(e)** 2D light transmission profile along radial and depth direction as simulated with the replicated model using a reduced spatial volume for spatial convolutions, which reproduces the observed underestimation in the original shown in (d). **(f)** Using an adequate spatial volume for spatial convolutions prevents the underestimation of light intensity present in the software underlying the published original model data (d) and our reproduction (e). Parametrization of simulations shown in this figure: Original model predictions were simulated using the original MATLAB software with the same optical fiber parameters and tissue properties as for our model, see Table 1. Other parameters of the original software were kept at their default: Resolution of the angular convolution: 32, time step of the multipath time integral: 5 fs, and the parameter max t : 5. Parametrization of our replication model was as summarized in Table 1 for the error-free prediction. For the reproduction of prediction errors of the original software, we reduced the volumes used for precalculation of the pencil beam and light cone before spatial convolutions as described in the text and adapted following parameters from their default (Table 1): xymax=400 μm, mu_tau = table1_Lutomirski.

Using the original study’s MATLAB software, we simulated light propagation with the original parametrization and compared the results to the published results. The simulations agree with the published results for the decay of light intensity along the entire depth (Fig 2a) and within small to intermediate radial distances (Fig 2b, 2c). At larger radial distances, the simulated light intensities noticeably underestimate published model estimates. This underestimation is not uniform across depth, which would only create a visually apparent edge in the 2D-profile, but instead shows a more pronounced underestimation at the ‘corners’ of the light profile (see arrow annotations in 2d). To reproduce these prediction errors, we simulated our replication model using the approximations by Lutomirski et al. for deriving the moments of time dispersion instead of the default parametrization (see Table 1), as our replication most closely resembled the original study’s model predictions (but not the experimental data) with this parametrization. We reproduced the underestimation along the edge when the spatial volume, in which the light cone was calculated, was radially limited to 400 μm. This limitation caused an underestimation for radii greater than 350 μm during final disk convolution, as the light cone volume was shifted by up to 50 μm to cover the fiber’s disk-shaped emission surface. Additionally, we reproduced the intensity underestimation at the ‘corners’ when the volume, in which the initial pencil beam was calculated, was limited to 500 μm radially and 700 μm along depth. This limitation led the interpolation of the pencil beam to exceed the precomputed volume during angular convolution to generate the light cone, see Fig 2e. Expanding the precomputed volumes before spatial convolutions removed these artificial boundary effects, see Fig 2f.

To prevent the prediction errors, our replicated code calculates the pencil beam within a volume large enough to accommodate the output dimensions defined by the parameters xymax and zmax during convolutions. The pencil beam volume is therefore expanded by ${\text{zmax}}_{\text{pencil}}=\sqrt{{\text{xymax}}^{2}+{\text{zmax}}^{2}}$ and ${\text{xymax}}_{\text{pencil}}=\text{xymax}\cos\theta_{\text{div}}+\text{zmax}\sin\theta_{\text{div}}$.

### Computing performance

To enhance computing performance of the replicated model, we employed vectorized computation using NumPy and, during calculation of the scattered component’s pencil beam, applied logarithmic sampling in the radial and multipath-time domain, extending the original approach, which only used radial logarithmic sampling. We also carried out angular convolution of the direct component in analytical form, avoiding the need for computation-intensive interpolation of this component during the final disk convolution. This enabled carrying out disk convolutions separately for the direct component at a finer and for the scattered component at a coarser step size. The finer resolution for the direct component prevented sampling artifacts near the emission surface, where it contributes strongly, while a coarser resolution for the smoother, and close to the emission surface, weaker scattered component saved computation time.

**Fig 3:**
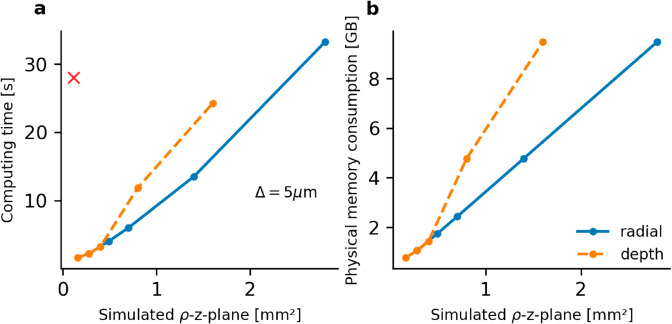
Computing speed and memory usage. **(a)** Computing time depending on simulated cortical volume, which for symmetry reasons, is represented by the plane spanned by radial and depth directions. Spatial resolution was Δ=5 μm. Computing time increases with increasing volume along radial (solid blue) and depth direction (dashed orange) with different slope. Original model computation time is marked with a red cross and was simulated in the same spatial volume as the first data point of the replicated model. Evaluation of the original model for different spatial volumes was not possible due to hard coding of these parameters in the published application. **(b)** Physical memory consumption depending on simulated plane in radial and depth direction. Color coding same as in (a). Memory consumption was recorded as the maximum resident size during simulation monitored with the Python built-in module resource. Parametrization of simulations shown in this figure: Light predictions with the original MATLAB software were simulated at the application’s default parametrization as described for the previous figure (Fig 2). Light predictions with the replicated model were simulated using the default model parametrization (Table 1) but for varying volume along radial direction changing the parameter xymax to [400,700,1000,2000,4000] μm with the maximum depth zmax=700 μm. Volume was varied along depth direction by changing the parameter zmax to [400,700,1000,2000,4000] μm, while setting the maximum radial distance to xymax=400 μm.

Our replicated model offers significantly improved computation performance compared to the original MATLAB software, as shown in Fig 3a. For a *ρ*-*z*-plane of $0.28\;{\text{mm}}^{2}$, representing a volume of $0.112\;{\text{mm}}^{3}$ along positive *x*, *y*, *z* coordinate values, our model runs in under 3 seconds as a single-threaded computation on a laptop with Intel Core i5-8265U CPU (1.60GHz × 8) processor and 16 GB RAM, compared to 28 s with the original MATLAB software. Computation of light in a $1\;{\text{mm}}^{3}$-volume along positive *x*, *y*, *z* coordinate values (being represented by a $0.98\;{\text{mm}}^{2}$ *ρ*-*z*-plane), is 47 times faster using our implementation compared to state-of-the-art Monte Carlo software [7], requiring 10 s as compared to 470 s of simulation time on the same hardware. Computing time scaled linearly with increasing simulation volume, enabling light predictions at distances up to 4 mm from the source in 30 s. When doubling the radial limit from 2 mm to 4 mm, execution time increased by a factor of about 2.5 (cf. Fig. 3a).

Due to high memory demands from NumPy vectorization, we monitored physical memory consumption via the maximum resident size using Python’s built-in resource package. Memory usage scaled linearly with simulated space, peaking around 10 GB for largest tested simulation volumes (max. radius and depth: 4 x $0.7\;{\text{mm}}^{2}$ and 0.4 x $4\;{\text{mm}}^{2}$), see Fig 3b.

### FAIR software implementation

The software has a GPL-3.0 license and adheres to the FAIR4RS guidelines [8], ensuring accessibility, re-usability, and openness. It is distributed as a Python package via PyPI (https://pypi.org/project/bsf-light/) for straightforward, cross-platform installation and requires only minimal dependencies (NumPy, SciPy, Matplotlib). Simulations are configured using a YAML-formatted parameter file, with default settings provided in defaults.yml. Users can run the simulation by executing run.py with file paths for the parameter file and the desired output location. The documented code, available at https://doi.org/10.5281/zenodo.15490385, facilitates modifications, for example for extending the simulation to new stimulator designs.

## Discussion

The advent of light-based manipulations and recording of neuronal activity has made computational prediction of light intensity essential in neuroscience research. Here, we replicated a previously closed-source light simulation model for cortical media, originally developed by Yona et al. (2016). This model achieves rapid and accurate predictions using a beam-spread function instead of the more computationally expensive Monte Carlo approach. The original model, however, is available only in a compiled and parameter-constrained format which produces results not fully consistent with the originally published data, making replication necessary to ensure transparency and the ability to apply the model in a broader set of contexts. In our replication, we documented the implementation, resolved ambiguities of the original model, corrected an error of the original software, and increased computational efficiency by an order of magnitude, enabling simulations in volumes larger than $1\;{\text{mm}}^{3}$ in around 10 s on standard laptop hardware. We published the light simulation software as an open-source tool along this article to promote reproducibility, allow others to build upon our work, and foster community-driven improvements.

### Generalization to other stimulator designs

The description of light spreading and absorption at the pencil-beam level enables generalization to various optical stimulator shapes. Here, we modeled an optical fiber with uniform emission of light across its angular direction, constrained by the fiber’s opening angle, and across its disk-shaped emission surface. This approach supports generalization to other stimulator geometries. Different optical fibers could be simulated by adjusting the parameters diameter and numerical aperture, while custom angular emission profiles and custom emission surfaces could also be implemented. A non-uniform angular emission profile would enter through the angular convolution integral, see Eq 14. Simulation of a non-disk-shaped emission surface would require adjusting the implementation of the spatial convolution to cover this surface instead of a disk, see Eq 17. Additionally, a non-uniform distribution of light emission along the emission surface could be accounted for in this integral.

### Limitations

This model assumes homogeneity of cortical tissue, characterized by a constant refractive index as well as uniform scattering and absorption coefficients. This simplification enables tractable simulations but limits applicability to bulk cortical tissue where spatial variations in optical properties and dynamic effects such as tissue heating are negligible. The strong agreement between model predictions and experimental data of blue-light (λ=473 nm) stimulation in mouse cortex suggests it captures key processes underlying light propagation in cortical tissue. However, model-fit to data may vary across varying species, cortical regions, or light wavelengths, and depending on the degree of tissue heating, which future work needs to explore. Applications of the model to cortex could be imprecise due to the varying level of scattering related to its laminar structure [9, 10]. While the model could be applied to determine the optical parameters of individually sliced cortical layers, it does not support modeling the multi-layer structure via layer-dependent parameters, as opposed to Monte-Carlo based tools [11]. Application of the model to subcortical structures may be more severely limited due to these structures’ more heterogeneous tissue organization, in which neuron types and densities often vary irregularly, for example, across functionally distinct nuclei of the thalamus [12], hypothalamus [13], and basal ganglia [14]. Further imprecision could arise from absorption of light by blood vessels, not being accounted for by the model, which is known to impact optogenetic stimulation outcomes [15].

Tissue heating predictions, which our model does not account for, are a critical concern for optogenetic stimulation interventions, as excessive heating can damage tissue near the stimulator. An alternative model that predicts tissue heating, although less computationally efficient, was recently introduced by Dong et al. [7]. Despite these limitations, the rapid computation enabled by our replication makes exploration of stimulator designs and model fitting more straightforward than with traditional Monte Carlo light simulations. Further, by sharing the source code openly, we open doors for potential improvements and expansion by the scientific community.

## References

[1] Yona G, Meitav N, Kahn I, Shoham S. Realistic numerical and analytical modeling of light scattering in brain tissue for optogenetic applications. eNeuro. 2016;3(1):ENEURO.0059-15.2015. doi: 10.1523/ENEURO.0059-15.2015 26866055 PMC4745178

[2] Emiliani V, Cohen AE, Deisseroth K, Häusser M. All-optical interrogation of neural circuits. J Neurosci. 2015;35(41):13917–26. doi: 10.1523/JNEUROSCI.2916-15.2015 26468193 PMC4604230

[3] Emiliani V, Entcheva E, Hedrich R, Hegemann P, Konrad KR, Lüscher C, et al. Optogenetics for light control of biological systems. Nat Rev Methods Primers. 2022;2:55. doi: 10.1038/s43586-022-00136-4 37933248 PMC10627578

[4] McLean JW, Freeman JD, Walker RE. Beam spread function with time dispersion. Appl Opt. 1998;37(21):4701–11. doi: 10.1364/ao.37.004701 18285927

[5] Lutomirski RF, Ciervo AP, Hall GJ. Moments of multiple scattering. Appl Opt. 1995;34(30):7125–36. doi: 10.1364/AO.34.007125 21060575

[6] van de Hulst HC, Kattawar GW. Exact spread function for a pulsed collimated beam in a medium with small-angle scattering. Appl Opt. 1994;33(24):5820–9. doi: 10.1364/AO.33.005820 20935985

[7] Dong N, Johnson E, Berlinguer-Palmini R, Zhong H, Dehkhoda F, Soltan A, et al. Optogenetic multiphysical fields coupling model for implantable neuroprosthetic probes. IEEE Access. 2024;12:129160–72. doi: 10.1109/access.2024.3441571

[8] Barker M, Chue Hong NP, Katz DS, Lamprecht A-L, Martinez-Ortiz C, Psomopoulos F, et al. Introducing the FAIR principles for research software. Sci Data. 2022;9(1):622. doi: 10.1038/s41597-022-01710-x 36241754 PMC9562067

[9] Wang H, Magnain C, Sakadžić S, Fischl B, Boas DA. Characterizing the optical properties of human brain tissue with high numerical aperture optical coherence tomography. Biomed Opt Express. 2017;8(12):5617–36. doi: 10.1364/BOE.8.005617 29296492 PMC5745107

[10] Chang S, Varadarajan D, Yang J, Chen IA, Kura S, Magnain C, et al. Scalable mapping of myelin and neuron density in the human brain with micrometer resolution. Sci Rep. 2022;12(1):363. doi: 10.1038/s41598-021-04093-y 35013441 PMC8748995

[11] Wang L, Jacques SL, Zheng L. MCML–Monte Carlo modeling of light transport in multi-layered tissues. Comput Methods Programs Biomed. 1995;47(2):131–46. doi: 10.1016/0169-2607(95)01640-f 7587160

[12] Hirai T, Nagaseki Y, Wada H, Shibazaki T, Matsumura M, Ohye C. Cell sparse zones in the ventrolateral thalamic mass in humans, monkeys and cats: their special reference to kinesthetic neurons. Appl Neurophysiol. 1985;48(1–6):316–23. doi: 10.1159/000101151 3837651

[13] Roberts DE, Killiany RJ, Rosene DL. Neuron numbers in the hypothalamus of the normal aging rhesus monkey: stability across the adult lifespan and between the sexes. J Comp Neurol. 2012;520(6):1181–97. doi: 10.1002/cne.22761 21935936 PMC4278435

[14] Oorschot DE. Total number of neurons in the neostriatal, pallidal, subthalamic, and substantia nigral nuclei of the rat basal ganglia: a stereological study using the cavalieri and optical disector methods. J Comp Neurol. 1996;366(4):580–99. doi: 10.1002/(sici)1096-9861(19960318)366:4<580::aid-cne3>3.0.co;2-0 8833111

[15] Azimipour M, Atry F, Pashaie R. Effect of blood vessels on light distribution in optogenetic stimulation of cortex. Opt Lett. 2015;40(10):2173–6. doi: 10.1364/OL.40.002173 26393692

